# Transcriptome analysis provides insights into the stress response crosstalk in apple (*Malus* × *domestica*) subjected to drought, cold and high salinity

**DOI:** 10.1038/s41598-019-45266-0

**Published:** 2019-06-21

**Authors:** Xingliang Li, Minji Li, Beibei Zhou, Yuzhang Yang, Qinping Wei, Junke Zhang

**Affiliations:** 1 Beijing Engineering Research Center for Deciduous Fruit Trees, Beijing Academy of Forestry and Pomology Sciences, Beijing, 100093 P.R. China; 2 Beijing Collaborative Innovation Center for Eco-environmental Improvement with Forestry and Fruit Trees, Beijing, 102206 P.R. China

**Keywords:** Abiotic, Transcriptomics

## Abstract

Drought, cold, and high salinity are three major abiotic stresses effecting apple tree growth and fruit production. Understanding the genetic mechanisms of crosstalk between stress responses signalling networks and identifying the genes involved in apple has potential importance for crop improvement and breeding strategies. Here, the transcriptome profiling analysis of *in vitro*-grown apple plants subjected to drought, cold and high salinity stress, showed a total of 377 upregulated and 211 downregulated common differentially expressed genes (DEGs) to all 3 stress treatments compared with the control. Gene Ontology (GO) analysis indicated that these common DEGs were enriched in ‘metabolic process’ under the ‘biological process’ category, as well as in ‘binding’ and ‘catalytic activity’ under the ‘molecular function’ category. Kyoto Encyclopedia of Genes and Genomes (KEGG) pathway analysis showed that common DEGs were mainly belong to the ‘biological functions’ category and 17 DEGs were identified in ‘environmental information processing’ sub-category which may act as signal transduction components in response crosstalk regulation. Overexpression of 5 upregulated genes individually, out of these 17 common DEGs in apple calli promoted the consistent upregulation of *DREB6*, *CBF1* and *ZAT10* and increased the mass weight and antioxidase ability, implying these five common DEGs involved in multiple pathways and improved comprehensive resistance to stress.

## Introduction

Plants are frequently exposed to various biotic and abiotic stresses, of which drought, cold and high-salinity stresses are the principal causes of declines in crop productivity worldwide^1^. Stress-inducing factors can occur simultaneously or sequentially and cause osmotic stress, water deficits, ionic imbalances, peroxidation damage and, ultimately, growth inhibition, as well as effects associated with similar physiological processes^2,3^. In response to these stresses, plants have developed diverse pathways that coordinate to combat and tolerate stress.

Many studies have revealed details of the signal transduction pathways that are activated by individual stresses^4^. For example, abscisic acid (ABA)-dependent and -independent pathways are activated by drought stress, the ICE-CBF-COR signalling pathway is activated by cold resistance, and the SOS pathway is induced by salt stress^5–7^. Moreover, similar regulatory components involving shared gene expression patterns, physiological indicators and phenotypic characteristics have been shown to be involved in drought, cold and high-salinity stresses. In some plant species, the induction of cold resistance also promotes drought resistance and high-salinity tolerance, which is consistent with an increase in the levels of osmo-regulatory compounds and antioxidant enzyme activities^8^. In other studies, an overlap between the expression patterns of stress-responsive genes in *Citrus*^9^, grape (*Vitis vinifera*)^10^, poplar (*Populus*)^11^, tea tree (*Camellia sinensis*)^12^, *Arabidopsis thaliana*^13^, maize (*Zea mays*)^14^, and other plant species was observed after drought, cold and high-salinity stress induction.

Overexpression of stress resistance genes involved in one type of stress can enhance resistance to other stresses, further suggesting complex cross-regulation of different stress signalling pathways. For example, the overexpression of *DREB2a*, which has been identified as a drought resistance gene, in transgenic *A. thaliana* and *Lotus corniculatus* forage plants resulted in enhanced tolerance to both drought and salt stress. Under stress conditions, the transgenic plants were taller and had longer roots, elevated levels of soluble sugars and a lower content of malondialdehyde compared with the control^15^. Antioxidase GPX3, commonly considered a vital scavenger of reactive oxygen species (ROS), also plays an important role in ABA-mediated stomatal closure under drought stress due to the oxidation of ABI1 and ABI2 by GPX3^16,17^. Previous studies have revealed genes and pathways that are involved in diverse abiotic stresses and that are potentially useful candidates for genetic engineering to improve multiple stress tolerance.

Transcriptome analysis has been widely applied to explore and identify differentially expressed genes (DEGs) involved in plant growth, fruit development and stress hormone regulation^18–20^. The availability of the apple draft genome sequence provides an opportunity for detailed analysis of stress resistance genes and their transcription; however, in contrast to *A. thaliana*^21,22^, maize^23^, and rice (*Oryza sativa*)^24^, little has been reported about transcriptome changes in apple in response to drought, cold and high salinity. Here, we describe RNA-seq analyses of apple plants grown under drought, cold or acute high-salinity stress to identify shared regulatory pathways, key functional genes or signal transduction components involved in the three stresses. This integrated study provides insights into the molecular mechanisms of the cross-regulation of abiotic stress responses in apples.

## Results

### Apple gene expression profiles in response to three abiotic stresses

After quality assessment and data filtering, an average of 8.16 billion reads with a Q30 >93.5% were retained as high-quality reads for each library (Supplementary Table 1). These clean reads were then combined in a *de novo* assembly, which resulted in 43,752 unigenes, of which 20,913 were longer than 1 kb (Supplementary Table 2).

**Figure 1:**
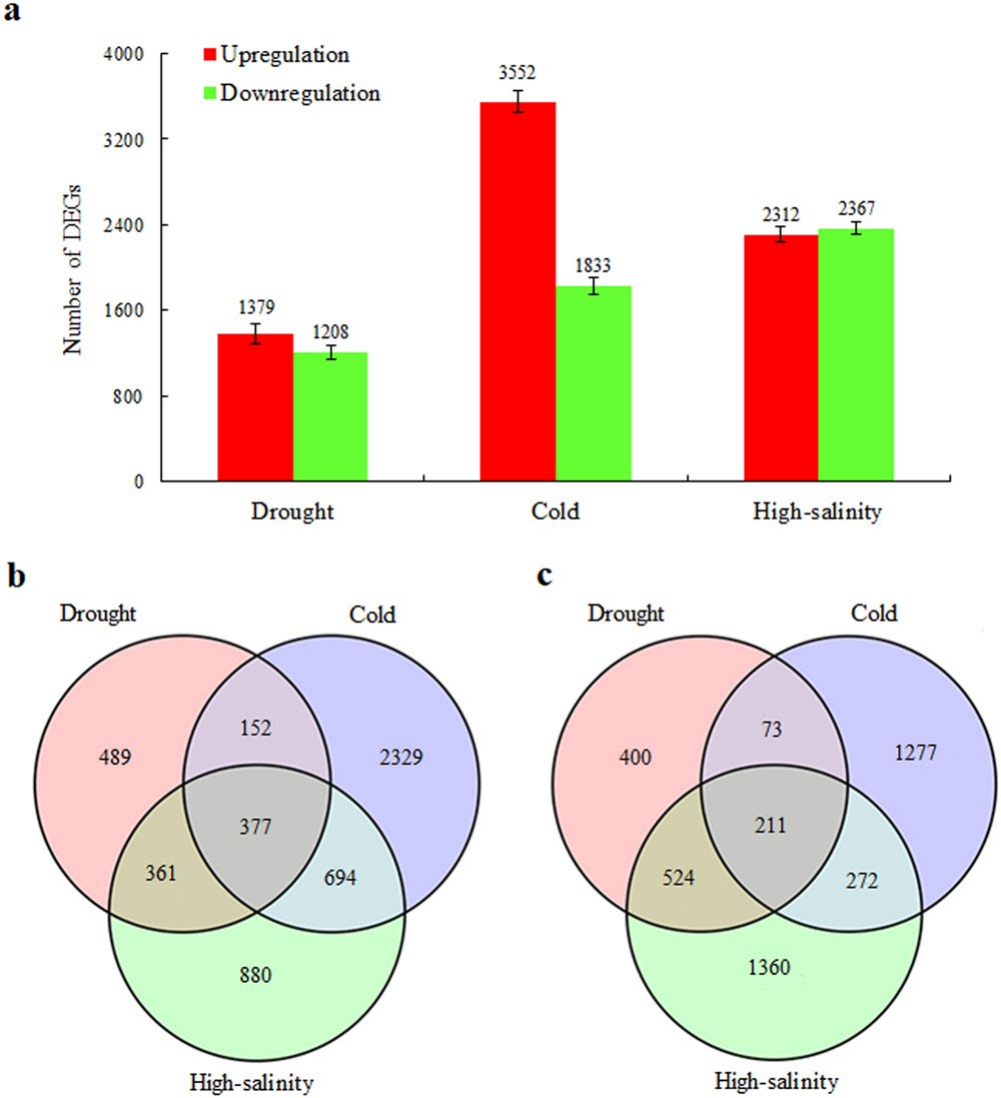
Apple gene expression profiles in response to drought, cold and high-salinity stress. (**a**) Total number of upregulated and downregulated genes. (**b**) Venn diagram of upregulated genes in the three samples vs. control. (**c**) Venn diagram of downregulated genes in the three samples vs. control.

To analyse variation in gene expression under drought, cold and high-salinity stresses, DEGs with FC > 2.0 and FDR < 0.01 between the treatments and controls (drought vs. control, cold vs. control, and high salinity vs. control) were identified (Fig. 1a). Generally, more DEGs were detected, including both upregulated and downregulated DEGs, in the cold and high-salinity treatment comparisons than in the drought vs. control comparison. Notably, there were 3552 upregulated DEGs under cold stress treatment, more than the number of upregulated DEGs in response to drought and high-salinity stress, with 1379 and 2312 members, respectively. Of the genes that were differentially expressed as a result of all three stresses (common DEGs), 377 were consistently upregulated and 211 were consistently downregulated (Fig. 1b,c).

### GO classification of common DEGs

To understand the functional categories of commonly expressed genes, Gene Ontology (GO) classification was performed on 377 commonly upregulated and 211 commonly downregulated genes (Supplementary Fig. 1). The most frequently assigned GO terms in the ‘biological process’ category were ‘metabolic process’ (GO: 0008152) and ‘cellular process’ (GO: 0009987); the most frequently assigned GO terms in the ‘cellular component’ category were ‘cell’ (GO: 0005623) and ‘cell part’ (GO: 0044464); and the most frequently assigned GO terms in the ‘molecular function’ category were ‘binding’ (GO: 0005488) and ‘catalytic activity’ (GO: 0003824) (Table 1).

**Table 1:** Gene Ontology (GO) classification of common differentially expressed genes (DEGs) in response to drought, cold and high-salinity stress.

Gene Ontology term	Number of DEGs	
	Upregulated	Downregulated
▶ Biological process		
biological regulation (GO:0065007)	43	16
cellular component organization or biogenesis (GO:0071840)	5	11
cellular process (GO:0009987)	59	39
developmental process (GO:0032502)	7	6
growth (GO:0040007)	1	2
localization (GO:0051179)	20	7
metabolic process (GO:0008152)	88	49
multi-organism process (GO:0051704)	1	—
multicellular organismal process (GO:0032501)	3	4
reproduction (GO:0000003)	1	1
reproductive process (GO:0022414)	5	3
response to stimulus (GO:0050896)	37	11
signalling (GO:0023052)	7	1
single-organism process (GO:0044699)	56	32
▶ Cellular component		
cell (GO:0005623)	38	24
cell junction (GO:0030054)	1	—
cell part (GO:0044464)	38	25
extracellular region (GO:0005576)	3	6
macromolecular complex (GO:0032991)	2	4
membrane (GO:0016020)	20	20
membrane part (GO:0044425)	11	12
membrane-enclosed lumen (GO:0031974)	1	—
nucleoid (GO:0009295)	1	—
organelle (GO:0043226)	32	13
organelle part (GO:0044422)	12	4
▶ Molecular function		
antioxidant activity (GO:0016209)	2	2
binding (GO:0005488)	83	32
catalytic activity (GO:0003824)	81	43
electron carrier activity (GO:0009055)	8	—
enzyme regulator activity (GO:0030234)	1	5
metallochaperone activity (GO:0016530)	1	—
molecular transducer activity (GO:0060089)	1	—
nucleic acid binding transcription factor activity (GO:0001071)	17	6
transporter activity (GO:0005215)	8	3

### Pathway analysis of common DEGs

**Figure 2:**
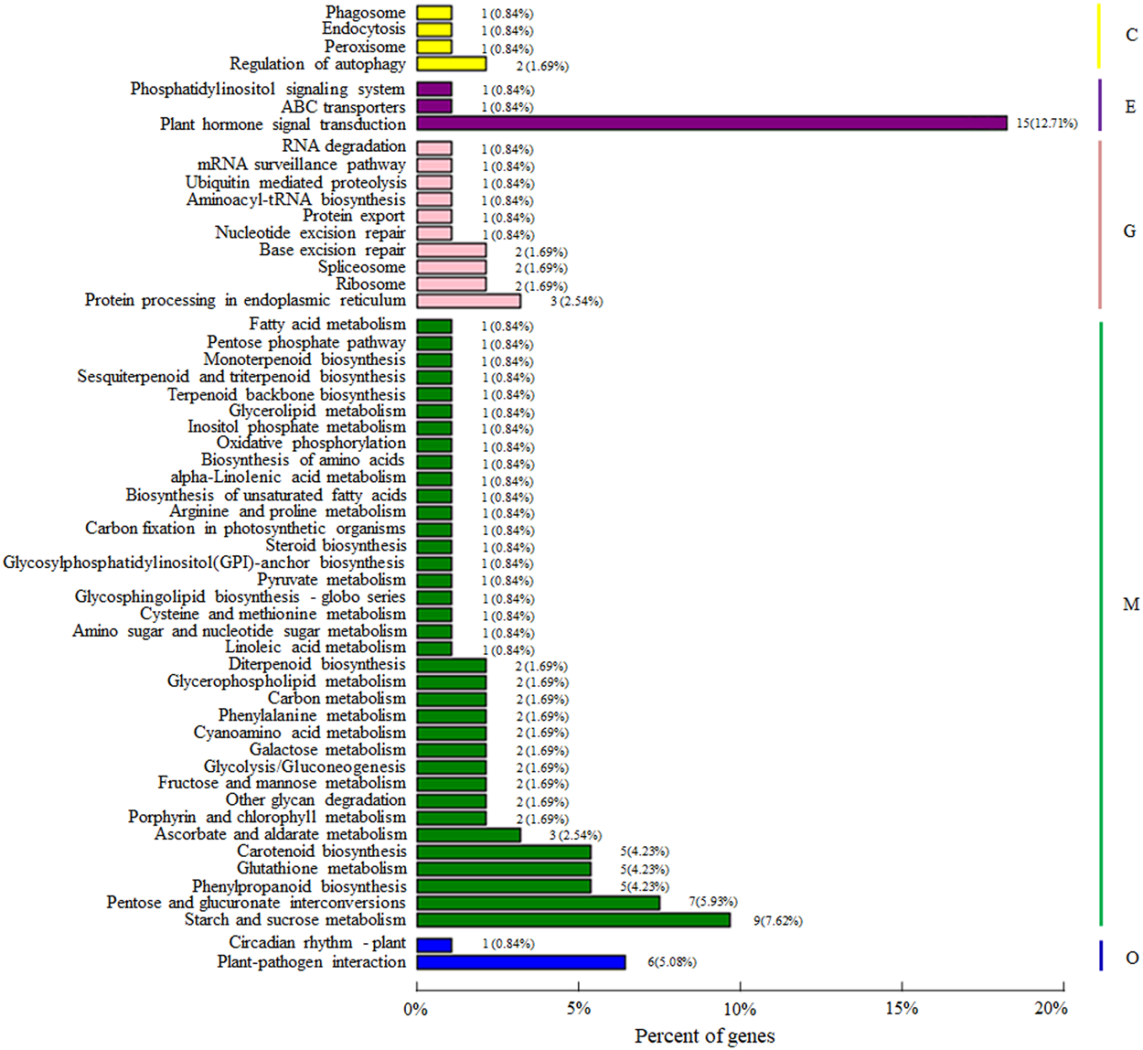
Kyoto Encyclopedia of Genes and Genomes (KEGG) pathway enrichment analysis based on the common differentially expressed genes (DEGs) involved in crosstalk between drought, cold and high-salinity stress regulation. C, cellular processes; E, environmental information processing; G, genetic information processing; M, metabolism; O, organismal systems.

To identify the DEGs putatively involved in crosstalk between drought, cold and high-salinity stress regulation, Kyoto Encyclopedia of Genes and Genomes (KEGG) pathway analysis was performed, and the biological functions of the common DEGs were further categorized (Fig. 2). The DEGs were mainly classified into ‘cellular processes’, ‘environmental information processing’, ‘genetic information processing’, ‘metabolism’, and ‘organismal systems’. Focusing on signal transduction components in pre-response crosstalk regulation, 17 DEGs in ‘environmental information processing’ were identified, including genes in the ‘phosphatidylinositol signalling system’, ‘ABC transporters’ and ‘plant hormone signal transduction’ pathways. ‘Plant hormone’ was the largest functional pathway, comprising a total of 15 hormone-related DEGs. Among the 17 DEGs, 15 were upregulated and 2 were downregulated by all three stresses, and some of these DEGs were classified as involved in the ABA, auxin, ethylene, and brassinolide (BR) hormone pathways. Seven of the 17 DEGs were members of the protein phosphatase 2 C family, which is predicted to play a role in the ABA regulation pathway (Table 2).

**Table 2:** Characters of shared differentially expressed genes (DEGs) between drought, cold and high-salinity stress responses classified in the ‘environmental information processing’ pathway.

No.	Accession number	Gene name	Function	Protein length (amino acids)	Common regulation
1	MD06G1200300	*DGK1*	diacylglycerol kinase	565	up
2	MD15G1010400	ABCB13	ABC transporter	479	down
3	MD01G1139200	*PP2C-37a*	protein phosphatase 2C	430	up
4	MD07G1203700	*PP2C-37b*	protein phosphatase 2C	423	up
5	MD07G1291000	*PP2C-51a*	protein phosphatase 2C	388	up
6	MD01G1220800	*PP2C-51b*	protein phosphatase 2C	354	up
7	MD15G1195800	*PP2C-56*	protein phosphatase 2C	443	up
8	MD02G1084600	*PP2C-77a*	protein phosphatase 2C	418	up
9	MD15G1212000	*PP2C-77b*	protein phosphatase 2C	446	up
10	MD07G1297400	*SAUR32*	auxin-responsive protein	134	up
11	MD12G1113400	*SAUR40*	auxin-responsive protein	149	up
12	MD05G1082000	*ABI5-5a*	ABA insensitive protein	431	up
13	MD15G1081800	*ABI5-5b*	ABA insensitive protein	419	up
14	MD13G1213100	*ERF1b*	ethylene-responsive transcription factor	229	up
15	MD06G1046300	*SAPK3*	serine/threonine-protein kinase	341	up
16	MD08G1102600	*BKI1*	BR receptor kinase inhibitor	326	down
17	MD11G1293900	*HPt3a*	histidine-containing phosphotransfer	152	up

ABA, abscisic acid; BR, brassinosteroids.

### Validation and expression correlation of common DEGs

**Figure 3:**
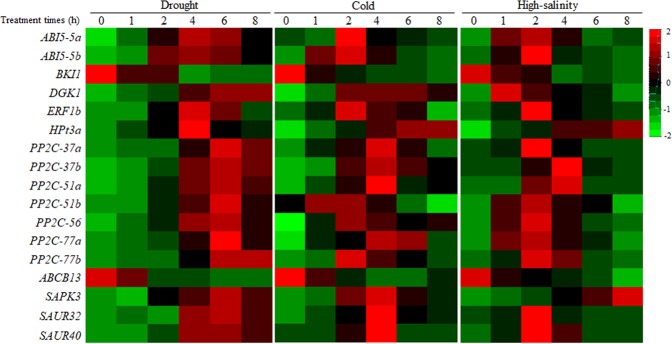
Expression patterns of common differentially expressed genes (DEGs) from drought-, cold- and high-salinity-stressed samples.

To validate the expression data obtained by RNA-seq, we performed qRT-PCR analysis of the expression of the 17 DEGs following the drought, cold and high-salinity treatments (primers are listed in Supplementary Table 3). In general, the data sets were congruent (Fig. 3). The expression of two downregulated genes, *BKI1* and *ABCB13*, was strongly inhibited at 4 h by all three stresses, while the 15 other genes were upregulated. Based on these results, five upregulated genes, *PP2C-37b*, *PP2C-77a*, *ABI5-5b*, *SAPK3*, and *HPt3a*, were selected for further analysis.

### Effect of overexpression of five common DEGs on stress resistance genes

**Figure 4:**
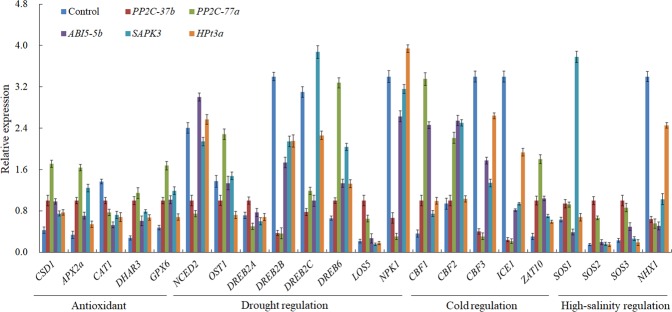
Effect of *PP2C-37b*, *PP2C-77a*, *ABI5-5b*, *SAPK3*, and *HPt3a* overexpression on regulatory genes involved in abiotic stress resistance. GenBank accession numbers: *PP2C-37b* (XM008373834.2); *PP2C-77a* (XM008390079.2); *ABI5-5b* (XM008379818.2); *SAPK3* (NM001320015.1); *HPt3a* (XM008361181.2).

To explore potential crosstalk between the regulatory pathways of the common DEGs, five upregulated genes were individually transformed into apple calli, resulting in a 6- to 15-fold increase in expression, as determined by qRT-PCR (Supplementary Fig. 2). A total of 22 genes in four response pathways (antioxidant, drought, cold and high-salinity response pathways) were used to reflect regulation by the common DEGs (primers are listed in Supplementary Table 4). The elevated expression of *PP2C-37b* coincided with the upregulation of four antioxidant enzymatic genes (*CSD1*, *APX2a*, *DHAR3*, and *GPX6*), three drought-related genes (*DREB2A*, *DREB6*, *LOS5*), two cold-related genes (*CBF1*, *ZAT10*), and three high-salinity-related genes (*SOS1*, *SOS2* and *SOS3*) (Fig. 4). Overexpression of *PP2C-77a* caused the upregulation of the above four antioxidant enzymatic genes, as well as three drought-related genes (*OST1*, *DREB6*, *LOS5*), three cold-related genes (*CBF1*, *CBF2*, *ZAT10*), and three high-salinity-related genes (*SOS1*, *SOS2* and *SOS3*). In comparison, overexpression of *ABI5-5b* promoted the expression of the *CSD1*, *APX2a, DHAR3* and *GPX6* antioxidant enzyme genes, *NCED2* and *DREB6*, which are related to drought stress, *CBF1*, *CBF2*, and *ZAT10*, which are related to cold stress, and *SOS3*, which is related to high-salinity stress. Furthermore, overexpression of *SAPK3* induced the transcription of the *CSD1*, *APX2a*, *DHAR3*, and *GPX6* antioxidant enzyme genes, *DREB2C* and *DREB6*, which are related to drought stress, *CBF1*, *CBF2*, and *ZAT10*, which are related to cold stress, and *SOS1*, which is related to high-salinity stress. Finally, overexpression of *HPt3a* caused the upregulation of *CSD1*, *APX2a*, *DHAR3*, *GPX6*, *DREB6*, *NPK1*, *CBF1* and *ZAT10*, while *CAT1*, *DREB2B*, *CBF3*, *ICE1* and *NHX1* were all downregulated in response to the overexpression of all five genes.

Overall, the regulation of common DEGs involved in drought, cold and high-salinity stress responses involved multiple pathways and complex regulation patterns within gene families, e.g., *PP2C-77a* promoted *CBF1* and *CBF2* expression but downregulated *CBF3*, while *SOS1* expression decreased and expression increased *SOS2* and *SOS3* in *ABI5-5b*-overexpressing calli. Of the 22 genes involved in stress resistance, the expression of three genes (*DREB6*, *CBF1* and *ZAT10*) consistently increased in response to the overexpression of all five common DEGs, implying cross-regulation downstream of those stress responses.

### Effect of the overexpression of five common DEGs on the stress resistance of apple calli

**Figure 5:**
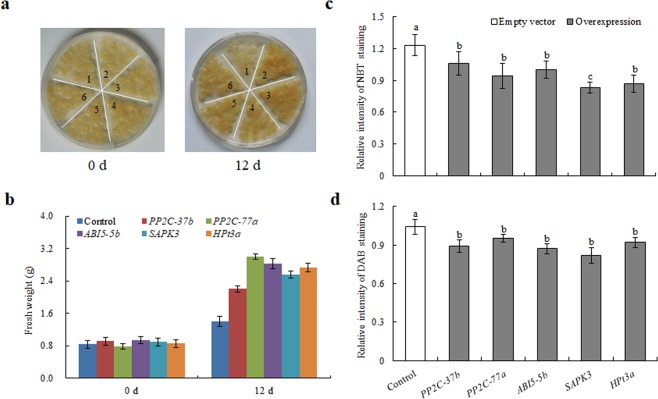
Effect of *PP2C-37b*, *PP2C-77a*, *ABI5-5b*, *SAPK3*, and *HPt3a* overexpression on the abiotic stress resistance of apple calli. (**a)** Apple calli transfected with an empty vector (1), *PP2C-37b*-overexpression calli (2), *PP2C-77a*-overexpression calli (3), *ABI5-5b*-overexpression calli (4), *SAPK3*-overexpression calli (5), and *HPt3a*-overexpression calli (6) were grown under 16 °C with 40% PEG8000 and 100 mM NaCl simultaneously. (**b)** Measurement of calli weight. Transfected apple calli were weighed before treatment (0 d) and after 12 d of treatment. (**c)** Relative intensity of NBT staining for transfected apple calli after treatment. (**d)** Relative intensity of DAB staining for transfected apple calli after treatment. Samples with different letters are significantly different *P* < 0.05.

To further examine the stress tolerance characteristics of these five common DEGs, transgenic apple calli were grown under 16 °C with drought and high-salinity stress simultaneously. After 12 days of cultivation, transgenic calli became dark yellow and gained more mass weight than the control (Fig. 5a,b). The *PP2C-77a* and *ABI5-5b* transgenic calli showed a better growth status than the other transgenic calli, indicating superior drought, cold and high-salinity stress resistance. In view of the upregulation of antioxidant enzyme genes, the transgenic calli with these five common DEGs were stained by nitroblue tetrazolium (NBT, for the detection of superoxides) and 3′,3′-diaminobenzidine (DAB, for the detection of H_2_O_2_) separately, and it was found that the overexpression of the five common DEGs promoted superoxide enzyme activities and decreased the H_2_O_2_ concentration (Fig. 5c,d). These results suggested that the elevation of antioxidant activity could be one pathway by which these five common DEGs confer comprehensive resistance to drought, cold and high-salinity stress.

## Discussion

In addition to horticultural attributes such as tree vigour, earlier flowering, fruit quality and yield, the ability of appropriate rootstocks to survive abiotic stresses, including drought, cold or salinization, in order to influence scion cultivars in apple cultivation has been widely recognized^25^. The apple dwarfing rootstock ‘SH6’, which was used in this paper, was bred by Ralls × Wuxiang crabapple in Shanxi province, China, in the 1980s. After decades of field evaluations, SH6 was retained not only because of its comprehensive agronomic dwarf traits, fruit sugar-to-acid ratio and stable production but also for, in particular, its multiple abiotic stress tolerance to drought, cold, high-temperature, and other stresses^26–28^, which greatly expanded apple cultivation areas and provided suitable research material for abiotic stress crosstalk regulation mechanisms.

Transcriptome analysis has been widely used in studies of apples, including investigations of flower development^29,30^, fruit ripening^31,32^, postharvest storage^33^, and biotic and abiotic stresses such as drought or low-temperature stress^34–37^. However, most studies have only investigated transcription profiles or mechanisms under a single stress. In this study, we focused on the expression genes involved in the physiological responses of apple seedlings to drought, cold and high-salinity stress and identified 1,379, 3,552 and 2,312 DEGs that were upregulated and 1,208, 1,833 and 2,367 DEGs that were downregulated by these stresses, respectively. Cold stress resulted in more DEGs than drought and high-salinity stress, which has also been reported for maize^23^ and wheat^38^ but was distinct from findings in *A. thaliana*, where cold resulted in less DEGs than drought and high-salinity stresses^19^. In addition, more DEGs were induced by drought than cold in cassava (*Manihot esculenta*)^39^, while fewer DEGs were induced by drought than cold in Dianthus spiculifolius^40^, and more DEGs were upregulated in response to drought than salinity stress in poplar^11^. The transcription response difference in the numbers of down- and upregulated genes among plant species might be related to the resistance levels and complexity of stress response pathways in these plants.

We found evidence of crosstalk between drought, cold and high-salinity stress signalling, with 377 commonly upregulated and 211 commonly downregulated genes. GO classification of these genes revealed that most commonly regulated DEGs were associated with the ‘biological process’ category (Supplementary Fig. 1) and that the three stresses primarily affected genes assigned ‘metabolic processes’ and ‘stress responses’ terms. Phytohormones have been proposed to play key roles in stress responses and/or adaptation^41^. In a KEGG pathway analysis, we found that the largest portion of the common DEGs were from the ‘plant hormone signal transduction’ pathway (15 members), with only two other response pathways with one DEG each in the ‘environmental information processing’ category (17 members) (Fig. 2). Among the 15 DEGs, ABA-, auxin-, ethylene-, and BR-related functions were observed in the early responses to drought, cold and high-salinity stress.

ABA has been reported to control the expression of drought, cold and high-salinity stress responsive genes, and most of the transcripts for genes encoding enzymes involved in ABA biosynthesis displayed enhanced expression under drought, oxidative, and temperature stresses^42^. Our results suggest crosstalk pathways or potentially genes involved in the responses to all three stresses involving ABA signalling, including the PP2C and ABI5 families. In plants, members of the PP2C family have been implicated as positive regulators within ABA-mediated signalling networks activated by diverse environmental stresses or developmental signalling cascades^43^. Moreover, the bZIP transcription factor ABI5 has been shown to bind to the promoter regions of many stress-responsive genes, thereby controlling the expression of ABA-inducible genes^17^. With 9 out of 15 common DEGs identified in this study being related to ABA signalling, our results add to the information on signalling cross-regulation in response to abiotic stress.

Five out of these 15 DEGs that were upregulated in response to all three stresses (protein phosphatases *PP2C-37b* and *PP2C-77a*, ABA insensitive transcription factor *ABI5-5b*, serine/threonine-protein kinase *SAPK3*, and histidine-containing phosphotransfer *HPt3a*) were overexpressed in apple calli. We found that the elevated expression of these common DEGs modulated the expression of multiple genes known to be involved in stress tolerance (Fig. 4). The common DEGs were predominantly associated with antioxidant-, drought-, cold- or high-salinity- stress regulation pathways: *PP2C-77a* overexpression caused increased expression of the *CSD1*, *APX2a*, *DHAR3*, and *GPX6* antioxidant enzymes and was involved in the drought-stress response through *OST1*, *DREB6*, *LOS5* regulation, the cold-stress response through *CBF1*, CBF2, and *ZAT10* upregulation, and the high-salinity-stress response through *SOS1*, *SOS2* and *SOS3* regulation. Comparatively, *ABI5-5b* overexpression caused upregulation of *NCED2* and *DREB6*, which were involved in drought regulation, *CBF1*, CBF2, *ZAT10*, which were involved in cold regulation, and only *SOS3*, which was involved in high-salinity regulation. The results from transgenic apple calli in response to drought, cold and high-salinity stresses also suggested these five common DEGs were involved in multiple pathways and improved comprehensive resistance to stress.

In addition, we also observed examples of the suppression of abiotic stress-inducible genes, such as *CAT1*, *DREB2B*, *CBF3*, *ICE1*, and *NHX1*, by these five common DEGs. The overexpression of protein phosphatases or transcription factors by common DEGs could directly or indirectly decrease the expression of relative abiotic stress genes, by which compensation regulation, especially in gene families, could occur. The consistency of the expression patterns of *CBF3* and its transcription factors *ICE1* and *ZAT10* and its putative target gene *CSD1* indicated that the multi-functionality of the five common DEGs might be upstream of the stress genes detected.

**Figure 6:**
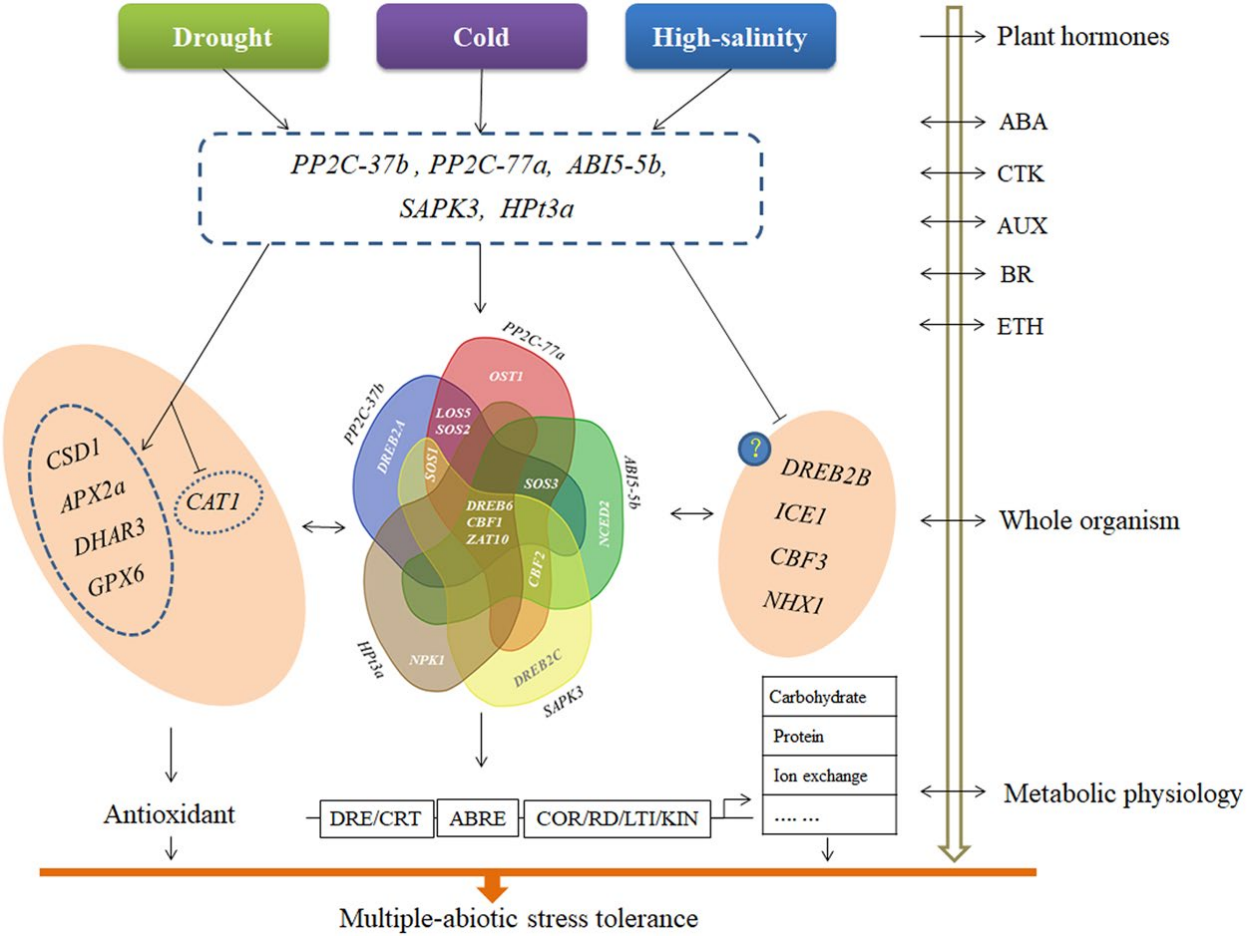
Crosstalk between drought, cold and high-salinity stress pathways linked to five common differentially expressed genes (DEGs) in apple. ABA, abscisic acid; CTK, cytokinins; AUX, auxin; BR, brassinolide.

As previous studies have shown, a systematic and organismal response occurs when plants suffer one or more bio/abiotic stress, mediated by Ca^2+^, ROS, phytohormones, and cellular, genetic or metabolic processing^44,45^. We only investigated the correlation of five co-upregulated genes with other well-identified resistance genes; however, the complexity of the regulatory networks was greater than expected (Fig. 6). The overexpression of these five DEGs promoted the expression of many genes in the antioxidant pathway, caused a decrease in *CAT1* expression and consistent increase in the expression of *DREB6*, *CBF1* and *ZAT10*, and had different regulatory effects on other genes. Positive functions on abiotic stress resistance genes, such as *ICE1* and *CBF3* for cold stress^46,47^ or *NHX1* for high-salinity stress^48^, were not well reflected under the overexpression of the five common DEGs, showing that specificity and coordination coexisted in multiple regulatory pathways.

Taken together, the comparative transcriptome analysis revealed 377 commonly upregulated and 211 commonly downregulated DEGs involved in the crosstalk between drought, cold and high-salinity stress in apple. GO classification and KEGG pathway analyses indicated that these common DEGs participate in various pathways related to signal transduction, antioxidants, metabolism, growth and developmental processes. Seventeen DEGs from the ‘environmental information processing’ pathway were selected for qRT-PCR analysis, five of which were overexpressed in apple calli and shown to regulate well-known genes and improve the abiotic stress resistance of transgenic calli. Overall, our study provided new insights into the crosstalk mechanism involved in drought, cold and high-salinity stress responses in apple and laid a reference for the investigation of other common DEGs for resistance breeding and functional studies.

## Materials and Methods

### Plant material and stress treatments

*In vitro* plantlets of the dwarf rootstock, *Malus* × *domestica* cv. ‘SH6’, were cultured on Murashige and Skoog (MS) medium supplemented with 1.0 mg/L 6-benzylaminopurine (6-BA), 0.4 mg/L 3-indolebutyric acid (IBA), 30 g/L sucrose and 7.0 g/L agar at 25 °C under a 16-h light/8-h dark photoperiod. After 15 days of rooting cultivation on MS medium (1/2 MS, 0.4 mg/L IBA), uniformly developing plants were transferred to stress treatment conditions. Drought and cold stress treatments were carried out with 40% PEG8000 and at 4 °C, respectively, as previously reported^49^. Plantlets cultured on the same MS medium, with or without 150 mM NaCl, were used for the high-salinity treatment and the control, respectively. All treatments were carried out for 4 hours, and leaves were harvested in three biological replicates and immediately frozen in liquid nitrogen.

Apple fruit calli (cv. Orin) were grown on MS medium supplemented with 0.5 mg/L indole-3-acetic acid (IAA) and 1.5 mg/L 6-BA at 25 °C in the dark and used for *Agrobacterium tumefaciens* infection, as described by Li *et al*.^50^. Transgenic calli were grown on the same medium at 16 °C supplemented with 40% PEG8000 and 100 mM NaCl for stress treatment.

### RNA extraction, library construction, and RNA-seq analysis

Total RNA from leaf samples of treated plantlets was extracted using the EASYspin plant RNA extraction kit (Biomed, China), and the integrity, concentration and purity of each sample were evaluated using 1% agarose gel electrophoresis and a NanoPhotometer spectrophotometer (IMPLEN, CA, USA). RNA-seq libraries were constructed according to the manuals provided by Illumina Inc. (San Diego, USA) and were sequenced on an Illumina HiSeq 2500 platform (Biomarker Technology Co.), which generated raw data of 150-bp paired-end reads. Mixed cDNA libraries from ten uniformly sized *in vitro* apple plants from each of the drought, cold, and high-salinity treatments and a control were used for RNA-seq.

### Mapping and annotation of sequencing reads

After quality assessment and data filtering, clean reads were obtained by removing the reads containing the adapter and low-quality reads, and these were then mapped to the *Malus* × *domestica* genome (GDDH13 Version 1.1) using HISAT2 software^51^ and *de novo* assembled using the paired-end method and StringTie software^52^. Subsequently, unigenes were functionally annotated using diverse protein databases, including the National Center for Biotechnology Information (NCBI) non-redundant protein (NR) database, the Swiss-Prot database, the Gene Ontology (GO) database, the Clusters of Orthologous Groups of proteins (COG) database, the EuKaryotic Orthologous Groups (KOG) database, the Protein Family (Pfam) database, and the Kyoto Encyclopedia of Genes and Genomes (KEGG) database. The gene coding sequences (CDS) were predicted with TransDecoder software (http://transdecoder.github.io).

### DEG analysis

Gene expression levels were analysed using fragments per kilobase of the transcript per million mapped reads (FPKM) method^53^. DESeq software was used to identify DEGs in pair-wise comparisons^54^, and the results of all statistical tests were revised to account for multiple testing with the Benjamini–Hochberg false discovery rate (FDR < 0.01). Sequences were determined to be significantly differentially expressed at a *P* value of <0.01 and a fold change (FC) of >2.0. DEGs shared between the three treatments were then analysed using KEGG pathway enrichment analysis, and the sequences of DEGs of interest were retrieved from the apple genome sequence (https://iris.angers.inra.fr/gddh13/).

### Genetic transformation of apple calli

To investigate the crosstalk between genes involved in multiple abiotic stress resistance, the complete CDS of one of the five selected common DEGs was inserted into the pCAMBIA1304 vector for overexpression and transformed into *A. tumefaciens* strain EHA105. The preparation of the infection suspension and overexpression of DEGs in apple calli were performed as previously described^55^.

### Quantitative real-time PCR (qRT-PCR)

Total RNA was extracted using the EASYspin plant RNA extraction kit (Biomed, China), and first-strand cDNAs were synthesized using M-MLV reverse transcriptase (Promega, USA) according to the manufacturers’ instructions. The 2 × SYBR-Green I RT-PCR Master Mix (Takara, Japan) was used as the labelling agent, and qRT-PCR was performed on a Bio-RAD C1000^TM^ Thermal Cycler with a CFX384^TM^ Real Time System (Bio-Rad, USA). The reaction mixture (10 µL) contained 5 µL 2 × Master Mix, 10 µmol·L^−1^ forward and reverse primers (0.5 µL each), 0.8 µL template cDNA, and 3.2 µL ddH_2_O. The PCR program was 95 °C for 1 min, followed by 40 cycles at 94 °C for 20 s, 60 °C for 20 s and 72 °C for 20 s. Three independent biological replicates were analysed for each sample, and β-Actin (XM008356922) was amplified along with the target genes as the internal control. A heat map was generated using custom scripts in R Project for Statistical Computing v3.3.1.

### Histochemical staining

Transgenic calli were stained with NBT for superoxide activity analysis and with DAB for H_2_O_2_ concentration detection in accordance with the methods of Hu *et al*.^56^. For NBT staining, 2 mM NBT (Sigma, Germany) solution was prepared in 20 mM phosphate buffer (pH 7.0). In addition, 1.0 mg/ml DAB (Sigma, Germany) was prepared in ddH_2_O and adjusted to pH 6.0 with Tris–HCl buffer (pH 7.5). Calli were incubated in NBT solution for 3 h and in DAB solution for 8 h separately in darkness and then kept in a solution of 3:1:1 ethanol:lactic acid:glycerol and photographed.

### Statistical analysis

All experiments were performed with at least three biological replicates and three technical replicates. Data are presented as the mean ± SD. Statistical differences between groups were evaluated using Duncan’s multiple-range test. *P* values < 0.05 were considered statistically significant.

## Data Availability

The datasets generated and analysed during the current study are available from the corresponding author on reasonable request.

## Supplementary information

Supplementary
