# Supplementary material for: Transcriptome analysis provides insights into the stress response crosstalk in apple (Malus × domestica) subjected to drought, cold and high salinity
Source: Sci Rep. 2019 Jun 21;9:9071. doi: 10.1038/s41598-019-45266-0 (PMC6588687; doi:10.1038/s41598-019-45266-0)
Supplement: Supplementary file 1 — Supplementary [file 41598_2019_45266_MOESM1_ESM.pdf]

# Supplementary Data

**Title:** Transcriptome analysis provides insights into the stress response crosstalk in apple (*Malus* × *domestica*) subjected to drought, cold and high salinity

**Authors:**
Xingliang Li[1,2], Minji Li[1], Beibei Zhou[1], Yuzhang Yang[1], Qinping Wei[1,2] & Junke Zhang [1Ψ]

**Address:**
[1]Beijing Engineering Research Center for Deciduous Fruit Trees, Beijing Academy of Forestry and Pomology Sciences, Beijing 100093, P.R.China.
[2]Beijing Collaborative Innovation Center for Eco-environmental Improvement with Forestry and Fruit Trees, Beijing 102206, P.R.China.

[Ψ]Author for correspondence, e-mail: zhangjk@nwsuaf.edu.cn)

**Competing financial interests:** The authors declare no competing financial interests.

**Supplementary Table 1.** Statistics for apple RNA-Seq libraries

| Library name | Clean reads | Clean bases | GC Content (%) | Cycle Q30 (%) |
|---|---|---|---|---|
| Control | 27,347,957 | 8,122,170,498 | 46.74 | 93.74 |
| Drought | 24,696,421 | 7,330,831,802 | 46.37 | 93.75 |
| Cold | 32,648,644 | 9,755,823,688 | 47.33 | 93.54 |
| High-salinity | 24,954,185 | 7,440,833,532 | 46.60 | 93.71 |
| Average | 27,411,801 | 8,162,414,880 | 46.76 | 93.69 |

**Supplementary Table 2.** Summary of Illumina transcriptome assembly for apple

| Library | Total Reads | Mapped Reads | Uniq Mapped Reads | Unigene | | |
|---|---|---|---|---|---|---|
| | | | | < 300bp | 300-1000bp | >1000bp |
| Transcript | 54,823,604 | 45,451,370 (82.90%) | 43,891,393 (80.08%) | 4165 (9.52) | 18674 (42.68) | 20913 (47.80) |

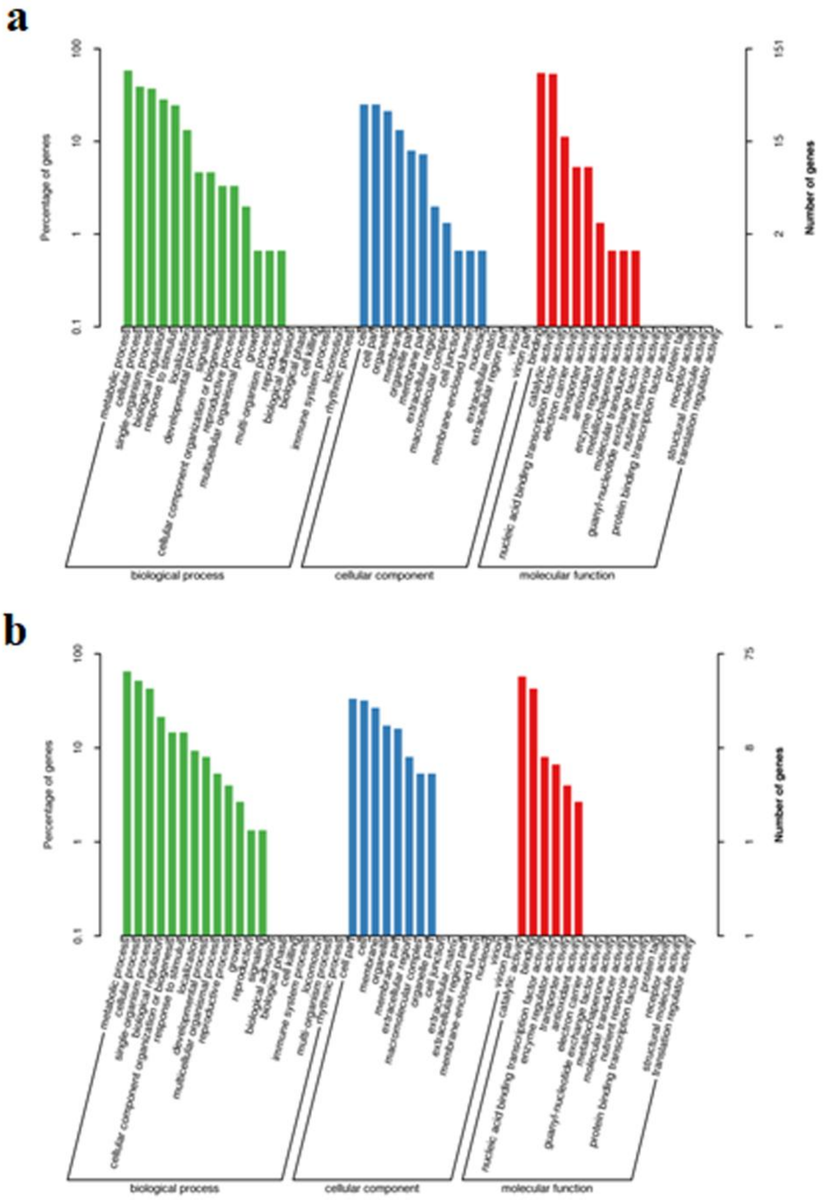


**Supplementary Figure 1.** The result of gene ontology (GO) classification for common differentially expressed genes (DEGs) involved in the crosstalk between drought, cold and high-salinity stress regulation. **a** Upregulated DEGs; **b** Downregulated DEGs.

**Supplementary Table 3.** Primers used for qRT-PCR of differentially expressed genes (DEGs).

| No. | Gene | Sequence No. | Primer Sequence (5' - 3') |
|---|---|---|---|
| 1 | *DGK1* | MD06G1200300 | Forward: GAGCCAGAGCAAGAAGTCCAAGC<br>Reverse: TGCTCCTCTTGAAACTGGGCTTA |
| 2 | *ABCB13* | MD15G1010400 | Forward: CGAGAACAGATGGGATTGGTAAG<br>Reverse: TCTCCTGCCTGAGTGTTGTAGC |
| 3 | *PP2C-37a* | MD01G1139200 | Forward: GGAACCCAACGGAAATCACT<br>Reverse: GACCTCCTCGTCCATCTTCA |
| 4 | *PP2C-37b* | MD07G1203700 | Forward: TTGTGGAAGATGATGCTGGTG<br>Reverse: ACACCGAAGTCATACCGAACC |
| 5 | *PP2C-51a* | MD07G1291000 | Forward: GGTCATCACTTGGAACGGAAAC<br>Reverse: CATCCTGGTCGGTTCGTTTG |
| 6 | *PP2C-51b* | MD01G1220800 | Forward: TGATGCGATTGATTGGGAGAC<br>Reverse: GCTTCCGTTCCAGTTGATGAC |
| 7 | *PP2C-56* | MD15G1195800 | Forward: TGCGTAGGGAGGAACAACAAG<br>Reverse: GCCAAAGAAATGAACGGGTG |
| 8 | *PP2C-77a* | MD02G1084600 | Forward: AGTGGATGCTGAGATTGGAGGAG<br>Reverse: TGCTACGATAATATGCGTTGGAC |
| 9 | *PP2C-77b* | MD15G1212000 | Forward: CGTGTCAAACTGTGGCGATTC<br>Reverse: CATCGCAAGAACACCGCAAAC |
| 10 | *SAUR32* | MD07G1297400 | Forward: GCTGAAAGACATCCCGAAAG<br>Reverse: TTGAACGGTGCGAAACTCCT |
| 11 | *SAUR40* | MD12G1113400 | Forward: ACTGGAGTCGTTGAGTAACCCTG<br>Reverse: TCTTCTTCCTCCTGCATGTTTG |
| 12 | *ABI5-5a* | MD05G1082000 | Forward: GGCAGTCTTCGGTGTACTCAT<br>Reverse: AGTCTCCTCAGCGGTCCCTAT |
| 13 | *ABI5-5b* | MD15G1081800 | Forward: CACTTTGGCTCCGAGTTCAGG<br>Reverse: AGGCACGGGTGACACGGATG |
| 14 | *ERF1b* | MD13G1213100 | Forward: GTTGAAAGAGTCCGCAAATCG<br>Reverse: CTTCAAACACCACCACATTCTC |
| 15 | *SAPK3* | MD06G1046300 | Forward: GATTTAGTGAAGACGAGGCAAGG<br>Reverse: AGCAATGTATGCAGGTGTTCCA |
| 16 | *BKI1* | MD08G1102600 | Forward: CTCCTCGCTCCTCCACCAACT<br>Reverse: GTCTCGTGTTTGCTTCCCTGA |
| 17 | *HPt3a* | MD11G1293900 | Forward: GCCGTGACTCAGTGGCAGAAG<br>Reverse: GCACCTATCCAGGCACTGCTA |
| 18 | *β-ACTIN* | XM008356922 | Forward: CTGAACCCAAAGGCTAATCG<br>Reverse: ACTGGCGTAGAGGGAAAGAA |

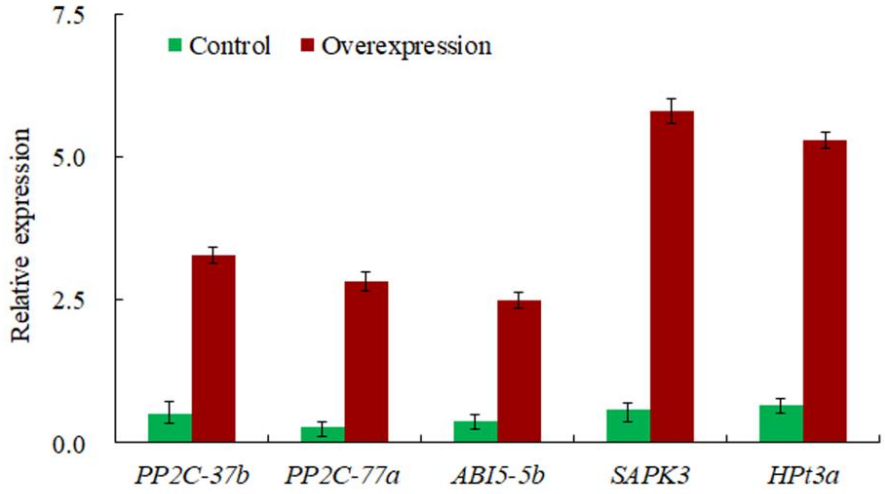


**Supplementary Figure 2.** Overexpression of five common differentially expressed genes (DEGs ) in apple calli. GenBank accession numbers: *PP2C-37b* (XM008373834.2); *PP2C-77a* (XM008390079.2); *ABI5-5b* (XM008379818.2); *SAPK3* (NM001320015.1); *HPt3a* (XM008361181.2).

**Supplementary Table 4.** Primers used for qRT-PCR of drought, cold and salinity related genes

| No. | Gene | Primer sequence (5’ - 3’) | GenBank Accession No. |
|---|---|---|---|
| 1 | CSD1 | Forward: CTGGGCCACACTTCAATCCTAA<br>Reverse: TAGAGGTATCTGCTTGTCAATAAGA | LOC103450984 |
| 2 | APX2a | Forward: GAAGCACTGTGCTCCGATAGTC<br>Reverse: GAATCTCAGGCCCTCCTGTAAC | LOC103413735 |
| 3 | CAT1 | Forward: CCGAGAGGTCCAATTCTGCTC<br>Reverse: CTCGAAGGAAGTCAGCACATGT | LOC103412104 |
| 4 | DHAR3 | Forward: CGTTTGTGCCAAGGCTTCCGTT<br>Reverse: CACAGGAACTTTACCTTCTGGGTA | LOC103444197 |
| 5 | GPX6 | Forward: CCATGCAACCAGTTTGGAGCC<br>Reverse: GAGAAGTTCCACTTGATGCTGTCA | LOC103446626 |
| 6 | *NCED2* | Forward: CTGAAACAGGGGACCTCAAA<br>Reverse: CGTAGCTAAGCGCGAAGAGT | KJ719311 |
| 7 | *OST1* | Forward: CTTGTTGGTGCTTATCCCTTTG<br>Reverse: CAGTGAAGATTCGGGAAAGGAG | KJ563286 |
| 8 | *DREB2A* | Forward: GAGCAGGCGAGATGGGAACT<br>Reverse: TCCCTTGCCTTTCATACAGC | XM008355947 |
| 9 | *DREB2B* | Forward: CCAGCCACAAACCCTAACTCA<br>Reverse: TTGGAGGTTCTTGCTTAACTTGG | XM008393437 |
| 10 | *DREB2C* | Forward: CACCAATCCCACAGCACAAAC<br>Reverse: GTGTCACCCACAACGACTAAACC | JQ669818 |
| 11 | *DREB6* | Forward: GCTTCTGTCGACGCCAAGCTTC<br>Reverse: TTAACTGAAGATCGAAGCCCAATC | GU732446 |
| 12 | *LOS5* | Forward: GATTCACCGTGGAAAGTTGG<br>Reverse: TGATTGGCAGTCTCGCTTG | XM008373811 |
| 13 | *NPK1* | Forward: CTCCCATTGTTATCATTCGTGTC<br>Reverse: TTGTCGCCCTTGGAAATTGGAAC | XM008387740 |
| 14 | *CBF1* | Forward: CTGAGGAGTCGGATGAGGTTTG<br>Reverse: CATAAGGGCACGTCAGCACAA | DQ074478 |
| 15 | *CBF2* | Forward: GAGTGTTGGAAAGCGGCGATGA<br>Reverse: GCCCTTCCGCCATACTATCCAG | KC603712 |
| 16 | *CBF3* | Forward: GGCTCGGAACTTATCCAACTG<br>Reverse: CCGACACTCCGCCAAACTCA | XM008363090 |
| 17 | *ICE1* | Forward: CAACACCGAGAACAAGGACGAG<br>Reverse: AGCAGCGAAGACAAGTTGGGAT | NM_001294038 |
| 18 | *ZAT10* | Forward: ACCACAGCCACTAAAGCAACA<br>Reverse: GAGCACTCGTGGGACCTTC | HM122498 |
| 19 | *SOS1* | Forward: CTCAAGAGGGTGCGGACGTGTC<br>Reverse: CCTTCAGCTATAACAACTCCACTC | XM_008352758 |
| 20 | *SOS2* | Forward: CAAAAGCACCATTCTCAAGCAC<br>Reverse: CCGACCAGCCAAAACCTCT | XM008364679 |
| 21 | *SOS3* | Forward: CTGGCTATGAGGACCCTGCGAC<br>Reverse: GATCGAACAAATTCTCCAAACTC | XM008378497 |
| 22 | *NHX1* | Forward: CCAGCACTGTCGTTTCACTCAC<br>Reverse: CTCGAGCTTTCGAACCCTGTCAC | XM008352393 |
